# Pseudogenes in Human Cancer

**DOI:** 10.3389/fmed.2015.00068

**Published:** 2015-09-25

**Authors:** Laura Poliseno, Andrea Marranci, Pier Paolo Pandolfi

**Affiliations:** ^1^Oncogenomics Unit, Core Research Laboratory, Istituto Toscano Tumori, Pisa, Italy; ^2^Institute of Clinical Physiology, Consiglio Nazionale delle Ricerche, Pisa, Italy; ^3^University of Siena, Siena, Italy; ^4^Cancer Research Institute, Beth Israel Deaconess Cancer Center, Departments of Medicine and Pathology, Beth Israel Deaconess Medical Center, Harvard Medical School, Boston, MA, USA

**Keywords:** pseudogenes, cancer, diagnostic markers, prognostic markers, ceRNAs, animal models of cancer, mutagenic factors

## Abstract

Recent advances in the analysis of RNA sequencing data have shown that pseudogenes are highly specific markers of cell identity and can be used as diagnostic and prognostic markers. Furthermore, genetically engineered mouse models have recently provided compelling support for a causal link between altered pseudogene expression and cancer. In this review, we discuss the most recent milestones reached in the pseudogene field and the use of pseudogenes as cancer classifiers.

## 1 Introduction

Since their discovery, pseudogenes have been neglected and considered bad copies of coding genes that have lost their coding potential and are void of function. Recently, it has emerged that pseudogenes represent a conspicuous part of the human transcriptome and proteome, as thousands of them are transcribed and hundreds are also translated (1, 2). Furthermore, it has been demonstrated that pseudogenes exert important coding-dependent and coding-independent functions that are involved in complex regulatory networks. It has also become apparent that pseudogenes contribute to the role that the non-coding genome plays in normal physiology as well as, when altered, in human disease. On this basis, pseudogenes are currently ranked among the classes of long non-coding RNAs (lncRNAs) (3–5).

**Figure 1:**
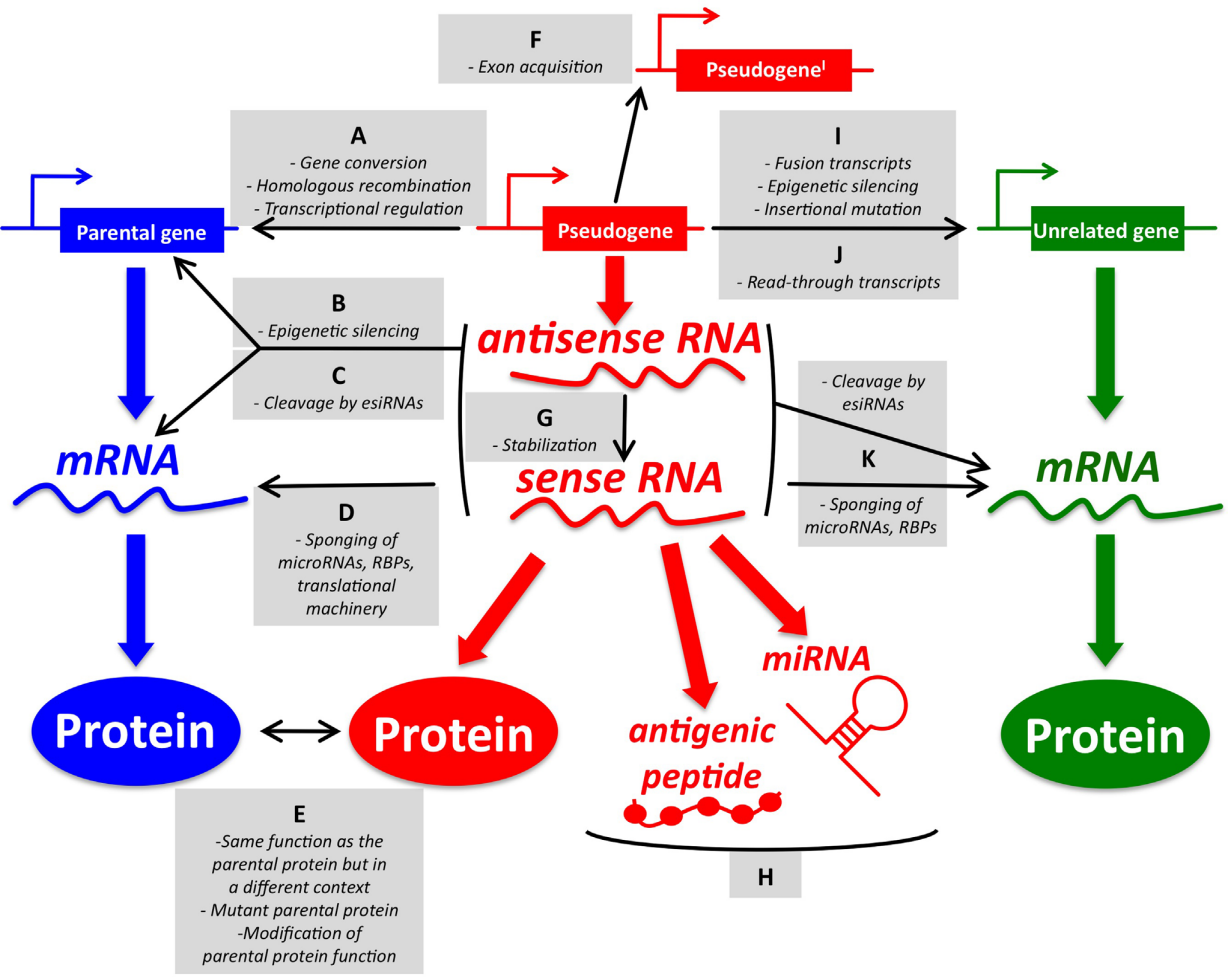
**An overview of pseudogene functions**. **(A–E)** Parental gene-related functions of pseudogenes (left). **(A)** Pseudogenes and parental genes can exchange genomic DNA through gene conversion or homologous recombination. The pseudogene promoter can also affect the transcription of the parental gene. **(B)** Pseudogene RNA transcribed in sense and antisense orientation can affect the transcription of the parental gene at the epigenetic level. **(C)** Pseudogene sense and antisense transcripts can form a double-stranded RNA that is cleaved into endogenous siRNAs. In turn, esiRNAs affect the parental gene expression at the post-transcriptional level. **(D)** Pseudogene RNA transcribed in sense orientation can compete with parental mRNA for the binding of microRNAs, RNA-binding proteins (RBPs), or the translational machinery. **(E)** Pseudogene proteins can be highly homologous to parental proteins, but be expressed in a different context (tissue, cellular compartment, pathophysiological condition). They can also carry gain-of-function mutations. Finally, they may affect the function of the parental proteins even if they are not fully functional. **(F–H)** Parental gene-unrelated functions of pseudogenes (center). **(F)** The *de novo* acquisition of new exons at either side (processed pseudogenes) or in the middle (non-processed pseudogenes) of their genomic sequence contributes to distinguishing the sequence, and hence the function, of pseudogenes compared to that of their parental genes. **(G)** Pseudogene RNA transcribed in antisense orientation can affect the stability of the sense pseudogenic transcript. **(H)** Pseudogene RNAs can be matured into microRNAs or translated into antigenic peptides. **(I–K)** Parental gene-unrelated functions of pseudogenes (right). **(I)** The “landing” of a processed pseudogene within other genes can produce several different scenarios. If the insertion site is an upstream intron, then the processed pseudogene will be cotranscribed with its host gene as a non-coding fusion transcript. If a protein is eventually translated, then it will most likely be short and contain only the pseudogene sequence. Pseudogenes inserted in upstream introns can also affect the transcription of the host gene by epigenetic silencing. If the insertion site is a more downstream intron, then the processed pseudogene will be cotranscribed with its host gene as a coding fusion transcript and the translated protein will be a chimera that is composed of both the gene and the pseudogene sequence. If the insertion site is in a 3′-UTR-expressing exon, then the fusion transcript will display an altered post-transcriptional regulation. If the pseudogene lands in a coding exon, the result will be insertional mutagenesis that will likely abrogate the expression of the host gene. **(J)** Pseudogenes and adjacent genes can be transcribed into joint read-through transcripts and translated into chimerical proteins. **(K)** Pseudogene RNAs working as source of esiRNAs or as sponges can also affect other unrelated genes besides the parental genes. The DNA/RNA/protein of a representative pseudogene, parental gene, and unrelated gene are shown in red, blue, and green, respectively. For a detailed overview of parental gene-related and unrelated functions of pseudogenes, please refer to Ref. (7) with updates reported in Ref. (9–18). For a list of the pseudogenes that function as sponges for microRNAs (a.k.a. competing endogenous RNAs) in cancer, please refer to Table 1.

While the origin of pseudogenes in the human genome and their role during evolution and speciation have been extensively studied for years (6–8), most of the known pseudogene functions have been discovered quite recently in the context of human cancer, where pseudogenic DNA, RNA, and peptides/proteins have been shown to exert parental gene-related and unrelated functions (for an overview, please refer to Figure 1).

In this review, we focus on recent advances in the field of pseudogenes in cancer, namely the establishment of their utility as diagnostic and prognostic factors, as well as the formal proof of their causal link with tumorigenesis, which has been established in genetically engineered mouse models.

## 2 Pseudogene Detection

Transcribed pseudogenes can be detected using RNA sequencing (RNA-seq), microarrays, and real-time PCR.

RNA sequencing (RNA-seq) allows to obtain an accurate assessment of all the pseudogene ­species ­present in a transcriptome and of their relative abundance. Crucially, it is also the only technique among the three that allows the discovery of new pseudogenes and hence it can provide the knowledge on which the other two techniques are built. However, RNA-seq costs are still quite high and *ad hoc* bioinformatic pipelines are required for data analyses. Fortunately, after the pioneering example published in 2012 by Kalyana-Sundaram et al. (9), quite a few pipelines of this sort have been published and have allowed for the definition of the pseudogene profile in multiple cancer types, including bladder cancer, breast cancer, cervical cancer, colorectal cancer, endometrioid carcinoma, gastric cancer, glioma, kidney cancer, lung cancer, melanoma, ovarian cancer, pancreatic cancer, and prostate cancer (5, 10, 19–22).

Microarrays, which offer the advantage of lower costs and easier data analysis compared to RNA-seq, are in fact rarely used for the purpose of pseudogene detection, unless they are designed to contain probes that bind specifically to pseudogenes and do not cross-bind to parental genes (23–25).

Finally, real-time PCR stands out for its low cost, high sensitivity, and high specificity. It is also the only technique among the three that is feasible to use routinely in the laboratory for a diagnostic or prognostic test. However, extreme care has to be taken in order to ensure that it is the pseudogene to be indeed amplified and the inadvertent amplification of the very similar parental gene is avoided. Multiple programs should be used in order to design primer pairs that recognize regions of low sequence similarity. It is also advisable to check that the amplified product is unique and that its size and sequence are the expected ones.

In the case that the pseudogene expression is assessed from the serum/plasma, it is also important to choose the right normalization control. Ideally, multiple genes need to be amplified by real time beforehand and then the one showing less variation among the samples under study should be chosen as control (26).

## 3 Pseudogenes as Diagnostic Markers in Human Cancer

Examples also exist that highlight the diagnostic power of specific pseudogenes. In gastric cancer, the levels of *SUMO1P3* pseudogene are up-regulated and can be used to differentiate patients with cancer from patients with benign gastric disease (27). Compared to healthy controls, patients with gastric cancer are also characterized by lower serum levels of *PTENP1* pseudogene, and such down-regulation, together with that shown by two additional lncRNAs (*CUDR* and *LSINCT-5*), has high diagnostic power [area under the curve (AUC) >0.8] (26). Analogously, hepatocellular carcinoma (HCC) patients are characterized by lower serum levels of the *INTS6P1* pseudogene compared to healthy individuals, and the diagnostic power of the pseudogene appears to be equal if not higher than that of alpha-fetoprotein, the most commonly used diagnostic biomarker for HCC (28).

Thanks to the development of the bioinformatic pipelines mentioned in Section 2 above, we have been able to fully appreciate the ability of aberrant pseudogenes expression to distinguish cancer tissues from normal tissues as well as one cancer subtype from another (9, 19, 21). Through the analysis of the RNA-seq data of 293 samples belonging to 13 cancer and normal tissue types, Kalyana-Sundaram and colleagues identified 218 pseudogenes that are expressed only in cancer samples and not in the correspondent benign tissues (9). Among them, the authors showed that 40 pseudogenes are cancer type-specific, being expressed only in a single cancer type (i.e., breast cancer, chronic lymphocytic leukemia, gastric cancer, pancreatic cancer, and prostate cancer). Finally, they focused on two pseudogenes with cancer subtype-specific expression: *ATP8A2-pg* (expressed in luminal but not in basal breast cancer samples) and *CXADR-pg* [mainly expressed in prostate cancer samples that do not carry an ETS fusion gene (29)].

Along a similar line, the analysis of RNA-seq data of 2,808 samples belonging to seven different cancer types [breast invasive carcinoma (BRCA), glioblastoma multiforme (GBM), kidney renal clear cell carcinoma (KIRC), lung squamous cell carcinoma (LUSC), ovarian serous cystadenocarcinoma (OV), colorectal carcinoma (CRC), and uterine corpus endometrioid carcinoma (UCEC)] has allowed Han and colleagues (19) to identify a plethora of pseudogenes that display subtype-specific expression. Namely, 48 in UCEC (endometrioid vs. serous), 138 in LUSC (basal, classical, primitive, and secretory), 71 in GBM (classical, mesenchymal, neural, and proneural), and 547 in BRCA (PAM50 subtypes: luminal A, luminal B, basal-like, Her2-enriched, and normal-like). Interestingly, this analysis confirmed that the expression of *ATP8A2-pg* is virtually absent in the basal-like breast cancer subtype. Furthermore, it showed that the pseudogene profile of UCEC samples can be used to accurately distinguish between the endometrioid and serous subtypes (AUC >0.9).

## 4 Pseudogenes as Prognostic Markers in Human Cancer

Once the correct cancer types and subtypes have been diagnosed, the choice of the best treatment option is further supported by the ability to foresee the prognosis. Recently, several examples have been reported that show how pseudogenes, besides being accurate diagnostic markers, are also valuable prognostic markers that can be used to stratify cancer patients on the basis of their life expectancy.

As an example, the *PTENP1* pseudogene functions as a ceRNA for its oncosuppressive parental gene *PTEN* (see below), and in clear cell renal cell carcinoma it has been shown that patients who do not express *PTENP1* display a shorter overall survival compared to those that do express *PTENP1* (30).

The *E2F3P1* pseudogene can present a guanine or an adenine at the rs9909601 SNP, and in HCC the overall survival of the patients carrying the GA/AA allele has been shown to be better than that of the patients carrying the GG allele (31).

The *OCT4* gene has multiple pseudogenes, which are preferentially expressed instead of the parental gene in human cancer cells (7, 11). Similar to what has been reported above for *E2F3P1*, *OCT4-pg1* (also known as *POU5F1P1* or *POU5F1B*) is a polymorphic pseudogene that presents an A or a G nucleotide at the rs10505477 SNP. Interestingly, gastric cancer patients who have undergone cisplatin-based chemotherapy and carry the GA/AA genotype have a poorer overall survival compared to those who have undergone the same treatment, but instead carry the GG genotype (32). Furthermore, Hayashi and colleagues (33) have shown that in gastric cancer *OCT4-pg1* is overexpressed due to a genomic amplification (the *OCT4-pg1* gene is located on chromosome 8q24 and is coamplified with its neighbor *c-MYC*) and that the OCT4-pg1 protein exerts an oncogenic role by promoting proliferation and angiogenesis while inhibiting apoptosis. In addition, the authors have observed that in stage IV patients, *OCT4-pg1* amplification is associated with a decreased overall survival.

Another OCT4 pseudogene, namely *OCT4-pg4*, promotes HCC cell proliferation by competing for miR-145 and hence sustaining the expression of its parental gene *OCT4* (see below for a ceRNA role of pseudogenes). Furthermore, it has been shown that *OCT4-pg4* expression levels stratify HCC patients according to their disease stage and overall survival, being that high expressors have a worse prognosis than low expressors (34).

Besides specific pseudogenes, a prognostic value has also been attributed to pseudogene signatures. Beginning with 183 glioma datasets belonging to the Chinese Glioma Genome Atlas (CGGA), Gao and colleagues (20) looked for pseudogenes that are associated with overall survival. In doing so, they identified a signature composed of six pseudogenes: *SP3P*, *ANXA2P3*, *PTTG3P*, *LPAL2*, *CLCA3P*, and *TDH*. In patients with shorter survival, the levels of the first five resulted higher, while those of *TDH* resulted lower. Next, the authors developed a formula that attributes a risk score to each glioma patient and is based on the cumulative expression levels of these six pseudogenes. By using this formula, they were able to classify CGGA patients according to their overall survival (patients with a high-risk score have a poorer prognosis than patients with a low-risk score). These findings were further validated in a different cohort of 350 glioma cases belonging to the Repository of Molecular Brain Neoplasia Data (Rembrandt) dataset.

In the same study mentioned in Section 3 above, the authors assessed the ability of pseudogenes to stratify KIRC patients according to their overall survival (19). This cancer type was chosen because, as of today, it is among the few that still lack reliable prognostic markers. By following a reverse approach compared to the one described in the paragraph above, the authors analyzed the expression profile of 446 KIRC samples and noticed that 500 pseudogenes are able to sort them into two distinct clusters. Interestingly, they observed that patients belonging to cluster 1 have a better prognosis than patients belonging to cluster 2 (19). A formula was developed that attributes a risk score to each KIRC patient and is based on the pseudogene expression levels as well as on other clinical variables. Interestingly, low-risk and high-risk patients can be stratified according to their overall survival using clinical variables alone. However, the low–medium risk group and the medium–high risk group can be distinguished only by the combination of clinical data and risk score based on the pseudogene expression levels. These results represent a paradigmatic example of the added value that pseudogenes can bring to prognostic predictions.

Finally, in the recently reported study by Ganapathi and colleagues (35), 21 primary and 24 recurrent high-grade serous ovarian cancer (HGSOC) samples were subjected to RNA-seq analysis. Twenty-one protein-coding genes and one non-coding gene were found to be differentially expressed between the two sample groups. The differential expression of the non-coding gene (the previously uncharacterized *SLC6A10P* pseudogene) was confirmed by real-time PCR in a larger validation cohort composed of 71 primary and 39 recurrent HGSOCs. Furthermore, low levels of *SLC6A10P* were found to be associated with longer time to progression, especially if considered together with high levels of one among the differentially expressed protein-coding genes (*COL2A1*) (35). This article offers a paradigmatic example of the ability of high-throughput techniques such as RNA-seq to point toward novel protein-coding and non-coding genes that not only contribute to our understanding of tumor pathogenesis but can also be exploited as useful biomarkers.

## 5 Pseudogenes as Mutagenic Factors

There are multiple lines of evidence in support of a causal link between altered pseudogene expression and the pathogenesis of human cancer (7, 36). First, pseudogenes often show cancer-specific deregulated expression, for example in the case of the OCT4 and NANOG pseudogenes, which are aberrantly expressed in cancer cells instead of their parental genes (7, 11, 18, 37). Second, similar to protein-coding genes, pseudogenes can undergo chromosomal rearrangements, amplification, deletion, and epigenetic silencing. HMGIY pseudogenes have been shown to be affected by chromosomal rearrangements in benign human tumors (38). *PTENP1*, the processed pseudogene of PTEN phosphatase, is a paradigmatic example of an oncosuppressive pseudogene that is deleted or hypermethylated in human cancer (23, 30, 39, 40). Conversely, as mentioned in the Section 4 above, the oncogenic *OCT4-pg1* is coamplified with *c-MYC* (33). Third, it has been reported that several variations in the sequence of pseudogenes such as those of PARP and CK2 can predispose to cancer (7).

Recently, a new and well-supported line of evidence for an oncogenic role of pseudogenes has been described. Cooke et al. (12) have shown that processed pseudogenes behave as mutagenic factors and impact the transcriptional landscape of the cells. Processed pseudogenes evolve from a retrotransposition event: the cDNA of the parental gene is retrotranscribed into DNA and is inserted randomly in the genome. As a consequence, pseudogenes do not contain introns, are located in a different region of the genome, and are subjected to a different regulation compared to that of their parental genes (7). The authors analyzed genome sequencing data of 660 cancer samples spanning 18 tumor types and identified 42 processed pseudogenes that are acquired somatically (namely, present in the cancerous tissue and absent in the matched normal tissue). These pseudogenes are mostly derived from highly expressed transcripts by LINE retrotransposition and are most frequently found in two cancer types: non-small cell lung cancer and colorectal cancer. In order to assess the consequences of pseudogenes retrotransposition on the expression of the pseudogenes themselves and of their host genes, the authors performed RNA sequencing on five samples in which they had identified 16 somatically acquired processed pseudogenes. Among them, 10 were found to have landed in intergenic regions, three in introns and three in exons. The last group of pseudogenes was further analyzed in light of the possible deleterious consequences on the expression of the host genes. The insertion of the *KRT6A* pseudogene in the 3′-UTR-expressing exon of the *MLL* gene was shown to cause the transcription of a fusion RNA, in which the 3′-UTR of *MLL* is replaced by the pseudogenic sequence. This, in turn, implicates that all the 3′-UTR-mediated post-transcriptional regulation of *MLL* expression is aberrantly lost. Analogously, the insertion of *KIF18A* pseudogene in the 3′-UTR-expressing exon of two overlapping transcripts (*KIAA1967* and *BIN3*) causes the transcription of two hybrid mRNAs in which the pseudogene sequence replaces that of the 3′-UTR of the host gene. Even more strikingly, the authors report that the landing of *PTPN12* pseudogene in the first exon of *MGA*, which is a likely oncosuppressor gene, causes an 8 kb deletion. This deletion spans the first exon itself, as well as the promoter region, and is ultimately responsible for the abrogation of *MGA* expression (12).

## 6 Pseudogenes as ceRNAs in Human Cancer

Pseudogene RNAs can behave as competing endogenous RNAs (ceRNAs). Because of their sequence similarity, the pseudogenes share multiple microRNA recognition elements (MREs) with their parental genes and can compete for the binding of common microRNA molecules (Figure 1D). As a consequence, pseudogenes sustain the expression of their parental genes and hence can acquire oncogenic or oncosuppressive functions when deregulated. For a comprehensive overview of ceRNA networks discovered in human cancer, both those that involve pseudogenes and other non-coding RNA classes, as well as those that involve protein-coding genes, please refer to Ref. (41–43) (these review articles also contain a detailed description of the rules that govern this class of regulatory networks and of the tools that are currently available to predict them).

The processed pseudogene *PTENP1* was the first ceRNA to be discovered in human cancer cells (23, 41). Since the discovery of *PTENP1*, the list of oncogenic and oncosuppressive pseudogenes that act as ceRNAs for their parental genes has been greatly expanded (those that have been characterized up to July 2015 are listed in Table 1). Furthermore, a multitude of pseudogene/parental gene pairs that show a positive correlation in their expression levels (which is strongly suggestive of ceRNA-based interactions) have emerged from the analysis of RNA-seq data of normal tissues (10) as well as from cancer cells such as breast and KIRC (19, 21).

**Table 1:** **Pseudogenes that function as ceRNAs for their parental genes or other genes in cancer**.

Pseudogene	Parental gene	Other genes	Shared microRNAs	Context	Reference
**Oncosuppressive pseudogenes**					
*PTENP1*	*PTEN*		miR-17, 19, 21, 26, and 214 families	Prostate cancer	(23)
				Melanoma	(39)
				Endometrial cancer	(44)
				ccRCC	(15)
				Hepatocellular carcinoma	(30)
				Gastric cancer	(45)
					(40)
*PTENP1*		*HRASLS5*	miR-135b	Breast cancer	(13)
*TUSC2P*	*TUSC2*		miR-17, 93, 299-3p, 520a, 608, and 661	Breast cancer	(46)
*INTS6P1*	*INTS6*		miR-17-5p	Hepatocellular carcinoma	(47)
**Oncogenic pseudogenes**					
*OCT4-pg4*	*OCT4*		miR-145	Hepatocellular carcinoma	(34)
*OCT4-pg5*	*OCT4*		miR-145	Endometrial carcinoma	(48)
*HMGA1P6*	*HMGA1*		miR-15, 16, 214, and 761	Thyroid carcinoma	(49)
*HMGA1P7*				Pituitary tumors	(50)
*CYP4Z2P*	*CYP4Z1*		miR-125a-3p, 197, 204, 211, and 1226	Breast cancer	(51)
*BRAFP1*	*BRAF*		miR-30a, 182, 590, and 876	DLBCL	(52)
*Braf-rs1*	*Braf*		miR-134, 543, and 653	Diffuse large B-cell lymphoma	(52)

Additionally, it has been shown that pseudogenes can act as ceRNAs not only for their parental genes but also for other genes (Figure 1K and Table 1). This is because the microRNAs that they share with their parental genes have also other targets and because they can be targeted by additional microRNAs. For example, *PTENP1* has been shown to exert oncosuppressive activity in prostate cancer cells that carry a deletion of the parental gene *PTEN* (23). Similarly, in breast cancer cells, it has been shown that *PTENP1* modulates the expression levels of *HRASLS5* (*HRAS-like suppressor family, member 5*) mRNA through the binding of miR-135b, a microRNA that has not been yet reported as *PTEN*-targeting (13).

Unitary pseudogenes represent an ultimate example of the ability of pseudogenes to exert ceRNA-based functions that are parental gene independent. The pseudogenes belonging to this class do not have parental counterparts because they derive from the progressive acquisition of mutations in protein-coding genes (7). Interestingly, Marques et al. (53) have shown that the function of unitary pseudogenes as ceRNAs can outlive their protein coding capacity, as indicated by the fact that the sequence of their MREs is more subjected to selective pressure than that of their coding sequence.

Lastly, besides the one pseudogene/one protein-coding gene cases, it is emerging that pseudogene-based ceRNA networks are extremely broad and pervade the cellular transcriptome as a whole. Furthermore, it has been shown that the loss or dysregulation of such networks is among the hallmarks of cancer cells compared to normal cells (13).

## 7 Animal Models of Pseudogenic ceRNAs

Besides the lines of evidence described in Section 5, formal proof of the causal link existing between pseudogenes and the pathogenesis of human cancer has recently come from genetically engineered mouse models (49, 52).

The article by Karreth et al. (52) focused on *Braf-rs1*, which is the processed pseudogene of the mouse Braf kinase, and shares 53 microRNA families with its parental gene (if both the coding sequence and the 3′-UTR are considered). This observation prompted the authors to evaluate if the pseudogene functions as a ceRNA. Indeed, they found that the overexpression of *Braf-rs1* in mouse NIH3T3 cells causes the up-regulation of Braf, the hyperactivation of the Erk pathway and, as a consequence, an increase in cell growth. Furthermore, the authors show that these effects are abolished when *Braf-rs1* is overexpressed in mouse cells that lack Dicer or that lack the parental gene (Braf KO cells), which suggests that the availability of mature microRNAs and the expression of Braf are required by *Braf-rs1* in order to exert its activity. Finally, the authors identified three microRNAs (miR-134, miR-543, and miR-653) as *Braf-rs1* and *Braf*-targeting and, by mutagenizing their MREs on *Braf* mRNA, proved that they are the mediators of the protective effects exerted by the pseudogene on the parental gene.

Next, the authors sought to investigate the consequences of *Braf-rs1* overexpression *in vivo*. To this end, a TRE-BPS transgenic mouse line, in which the ubiquitous expression of *Braf-rs1* is under the control of a doxycycline (dox)-inducible Tet-response element (TRE), was generated. This line was crossed with the CAG-rtTA line, and the compound transgenic animals were placed on a dox-containing diet at 3 weeks of age. The effects of the induction of *Braf-rs1* expression were dramatic: after approximately 4 months of treatment, the mice started to die and their median survival was as short as 400 days. Moribund mice were characterized by splenomegaly and enlarged lymph nodes, all symptoms that an in-depth flow cytometric analysis revealed to be the result of an aggressive form of diffuse large B-cell lymphoma (DLBCL).

*Braf-rs1*-induced lymphomas were further characterized in multiple ways. First, lymphoma cells obtained from the spleen of TRE-BPS/CAG-rtTA mice were transplanted into immunocompromised NSG mice, where they were shown to cause splenomegaly and to infiltrate all of the tested organs, which indicates that they are transplantable and highly aggressive. Furthermore, if doxycycline was removed once the splenomegaly became apparent, the lymphomas largely regressed, indicating that the tumors are markedly addicted to *Braf-rs1*, and its aberrant expression is required for tumor maintenance. Second, histological analyses proved that, consistent with the ceRNA hypothesis, *Braf-rs1*-driven tumors are indeed characterized by increased Braf and pErk levels. Furthermore, when transplanted NSG mice were treated with a Mek inhibitor, a marked impairment of the ability of lymphoma cells to infiltrate other organs was observed, which indicates that the tumors are addicted to *Braf-rs1* because they are addicted to the Erk pathway. Finally, the comparison of three distinct transgenic lines (TRE-BPS, TRE-BPS^CDS^, and TRE-BPS^3′-UTR^) allowed to establish that the aberrant expression of the 3′-UTR of *Braf-rs1* is sufficient to cause an increase in Braf levels, the hyperactivation of the Erk pathway, and a phenotype that is very similar to that caused by full-length *Braf-rs1*. On the contrary, the overexpression of *Braf-rs1* coding sequence does not cause a marked increase in *Braf* levels and induces a milder phenotype. These results provide further confirmation that *Braf-rs1* is a non-coding ceRNA that exerts an oncogenic function by increasing *Braf* levels.

These genetically engineered mouse models represent a proof of principle that aberrantly expressed pseudogenes are necessary and sufficient to cause cancer by working as ceRNAs for their oncogenic parental genes. Furthermore, even if the study is based on the mouse Braf pseudogene, it is of relevance to human cancer for two reasons.

On the one hand, in the same study, Karreth et al. (52) show that *BRAFP1*, the processed pseudogene of human BRAF, is involved in a ceRNA-based relationship with its parental gene as well and, for this reason, has oncogenic potential. The microRNA families shared between *BRAF* and *BRAFP1* were found to be 40, while miR-30a, miR-182, miR-590, and miR-876 were formally demonstrated as *BRAF* and *BRAFP1*-targeting. In addition, *BRAFP1* genomic locus was found amplified in the vast majority of cancer types featured in TCGA, including DLBCL, where, as expected by ceRNA partners, *BRAFP1* and *BRAF* expression levels show a positive correlation. In light of this correlation, and of the mouse phenotype described above, DLBCL cell lines were chosen for a further examination of the ceRNA-based effects of *BRAFP1* on BRAF. Analogously to the effects exerted by *Braf-rs1* overexpression on Braf (see above), *BRAFP1* overexpression was shown to exert a protective role on BRAF levels. Conversely, the down-regulation of *BRAFP1* by shRNA caused a reduction in BRAF and pERK levels and was accompanied by a decrease in cell proliferation.

On the other hand, it has been recently shown that human *BRAF* exists as two transcript variants, which differ in their 3′-UTRs (54). The canonical variant, which carries the reference 3′-UTR reported in all the databases, is up to 0.6 kb long and is highly homologous to the 3′-UTR of *BRAFP1* pseudogene [>90% sequence identity, as shown in (54)], while it is completely unrelated with the 3′-UTR of mouse *Braf*. Conversely, the X1 3′-UTR variant, which differs from the canonical in length (up to 7 kb) and in sequence (it is derived from the alternative splicing of an extra 19th *BRAF* exon), is highly homologous to mouse *Braf* 3′-UTR (84% sequence identity, according to https://blast.ncbi.nlm/). This in turn means that, although only the canonical *BRAF* can benefit from the protective effects of *BRAFP1*, total *BRAF* levels may be subjected to the regulation not only of the canonical *BRAF*-targeting microRNAs but also of the mouse *Braf*-targeting microRNAs. Interestingly, among the 191 MREs (for 100 microRNA families) that are predicted along the mouse *Braf* 3′-UTR according to Karreth et al. (52), 74 MREs (for 58 microRNA families) are also present in the 3′-UTR of human *BRAF-X1* (XM_005250045.1) (Table S1 in Supplementary Material).

Together these results support the notion that, analogously to *PTEN* tumor suppressor (41), also *BRAF*, with its transcript variants and its pseudogenes, is under tight microRNA-mediated regulation and is likely involved in complex ceRNA-based networks. This in turn suggests that at the basis of the oncogenic potential of such a powerful protein kinase, there might be not only the widely known and extensively studied mutation at the Val600 residue but also an aberrant gene expression (55).

## 8 Future Perspectives: Pseudogenes as Cancer Classifiers

Personalized medicine in cancer treatment is based on the assumption that every person harbors a unique variation of the human genome in the cancer that he/she develops and should be treated accordingly. Thus, in order for a personalized treatment to be effective, it is crucial to achieve a detailed classification of the cancer genome and epigenome. To this end, a classification solely based on the tissue of origin and on pathological features has shown its limitations. Conversely, large-scale projects that have merged the output of multiple omics techniques (whole-exome sequencing, DNA copy number variations, DNA methylation, mRNA-seq, microRNA-seq, and proteomics) have shown the power of a classification based on molecular alterations. Such an approach allows the subdivision of virtually every cancer type into multiple subtypes and offers guidance in the treatment of each patient with the drug or drug combination that has the highest chance to be effective. Furthermore, it shows that molecular level similarities exist among cancer types of different tissue of origin and offers the rationale for proposing the use of non-standard therapeutic strategies (5, 56).

Within this scenario, and in light of the fact that the diagnostic and prognostic power of pseudogenes is very high [in some cases even higher than that of microRNAs and mRNAs (19)], it is not surprising that it has been suggested to include RNA-seq of pseudogenes and, in more general terms, of lncRNAs, among the “omics” that are used to perform molecular classifications (57). The possibility to exploit such accurate markers of cell identity could represent a crucial refinement and a further step toward an effective personalized medicine. If this is the case, the rescue of the pseudogenome from the genetic junk will be complete and hopefully will pave the way for the establishment of the causal link existing between other classes of non-coding RNAs and human cancer.

## Conflict of Interest Statement

The authors declare that the research was conducted in the absence of any commercial or financial relationships that could be construed as a potential conflict of interest.

## Supplementary Material

The Supplementary Material for this article can be found online at http://journal.frontiersin.org/article/10.3389/fmed.2015.00068
