# Supplementary material for: Pseudogenes in Human Cancer
Source: Front Med (Lausanne). 2015 Sep 25;2:68. doi: 10.3389/fmed.2015.00068 (PMC4585173; doi:10.3389/fmed.2015.00068)
Supplement: Supplementary file 1 [file Table_1.PDF]

Supplementary Table 1. MREs shared by *Braf*, *Braf-rs1* and *BRAF X1* 3'UTRs.

	microRNA family	seed match start	seed match end	rs1	X1
1	let-7/98/4458/4500	6045	6051		X
2	miR-103a/107/107ab	2881	2887	X	
	miR-103a/107/107ab	3194	3200		X
	miR-103a/107/107ab	7160	7166		X
3	miR-124/124ab/506	6697	6703		X
4	miR-125a-3p/1554	5935	5941		X
5	miR-128/128ab	6603	6610		X
6	miR-129-5p/129ab-5p	8069	8075		X
	miR-129-5p/129ab-5p	9273	9279		X
7	miR-130ac/301ab/301b/301b-3p/454/721/4295/3666	6826	6832		X
8	miR-133abc	8362	8369		X
9	miR-134/3118	3405	3411	X	X
10	miR-136	9284	9290		X
	miR-136	9525	9531		X
	miR-136	9696	9702		X
11	miR-137/137ab	6710	6716		X
	miR-137/137ab	8457	8463		X
12	miR-139-5p	8011	8017		X
13	miR-142-3p	3827	3834		X
14	miR-146ac/146b-5p	9194	9200		X
15	miR-149	6585	6591		X
16	miR-153	3868	3874		X
17	miR-15abc/16/16abc/195/322/424/497/1907	2878	2884		X
18	miR-17/17-5p/20ab/20b-5p/93/106ab/427/518a-3p/519d	3026	3032	X	
19	miR-181abcd/4262	3501	3507	X	X
20	miR-18ab/4735-3p	6538	6544		X
21	miR-196abc	3700	3706	X	X
22	miR-19ab	4063	4070		X
23	miR-1ab/206/613	9124	9130		X
24	miR-214/761/3619-5p	4893	4900		X
25	miR-217	9068	9074		X
26	miR-218/218a	6901	6907		X
27	miR-221/222/222ab/1928	3354	3361	X	X
28	miR-223	3490	3496	X	
29	miR-23abc/23b-3p	3503	3509	X	X
	miR-23abc/23b-3p	9656	9662		X
30	miR-25/32/92abc/363/363-3p/367	6709	6715		X
	miR-25/32/92abc/363/363-3p/367	6801	6807		X
31	miR-27abc/27a-3p	6604	6610		X
	miR-27abc/27a-3p	8480	8486		X
32	miR-28-5p/708/1407/1653/3139	5294	5300		X
33	miR-290-5p/292-5p/371-5p/293	5467	5473		X
34	miR-296-3p	4047	4053		X
35	miR-300/381/539-3p	9486	9492		X
36	miR-30abcdef/30abe-5p/384-5p	8195	8202		X
	miR-30abcdef/30abe-5p/384-5p	8634	8640		X
37	miR-31	2997	3003	X	
38	miR-320abcd/4429	5988	5994		X
	miR-320abcd/4429	6414	6420		X
39	miR-326/330/330-5p	6587	6593		X
40	miR-338/338-3p	3767	3773		X
41	miR-33a-3p/365/365-3p	6178	6185		X
42	miR-33ab/33-5p	2675	2681	X	X
43	miR-342-3p	3505	3511	X	X
44	miR-34ac/34bc-5p/449abc/449c-5p	9142	9148		X
45	miR-374ab	9605	9611		X
46	miR-376c/741-5p	9645	9651		X
47	miR-378/422a/378bcdefhi	6963	6969		X
48	miR-382	3667	3673	X	X
49	miR-448/448-3p	3867	3874		X
50	miR-486-5p/3107	3535	3541	X	X
	miR-486-5p/3107	6630	6636		X
51	miR-491-5p	5942	5948		X
52	miR-494	9091	9097		X
53	miR-495/1192	2614	2621	X	X
	miR-495/1192	3653	3659		X
54	miR-539/539-5p	3300	3306	X	X
	miR-539/539-5p	9472	9478		X
55	miR-542-3p	6420	6426		X
56	miR-543	2731	2737	X	X
57	miR-544/544ab/544-3p	6733	6739		X
58	miR-590-3p	3642	3648	X	X
	miR-590-3p	9276	9282		X
59	miR-653	2714	2721	X	
	miR-653	2774	2780	X	X
60	miR-7/7ab	3400	3406	X	X
	miR-7/7ab	3809	3815		X
61	miR-96/507/1271	6680	6686		X
	miR-96/507/1271	9144	9150		X
		TOTAL		20	74

The table lists the MREs (seed matches) that are predicted in the 3'UTR of mouse *Braf* (NM_139294.5) and are shared by the 3'UTR of *Braf-rs1* pseudogene (Gm18189) and/or the 3'UTR of human *BRAF X1* transcript variant (XM_005250045.1). The seed matches that are shared by both *Braf-rs1* and *BRAF X1* are 16.
